# 

**Published:** 1887-04-23

**Authors:** 


### CONTENTS.

**Kilts and Physic** - 51

**Words of Consolation.**—XXX. Death - 52

**Books, Good and Otherwise** - 52

**Nurse Marion's Romance.** By Kate Furley. Chapter V. Complications - 53

**Moral and Physical Massage.** By Dr. Stretch Dowse - 55

**Miss Manson's Paper**—Two Protests - 57

**Notes and News.**—Appointments.—Miscellaneous Items.—The French Protestant Hospital.—Newton Cottage Hospital Report.—Vacancies.—Curious Advertising.—The late Duchess of Norfolk.—Donations, Legacies, etc., to Medical Charities, etc., etc. - 58

**Annotations.**—The King of the Belgians at the London Hospital.—Admission and Discharge of Patients.—Religion in French Hospitals - 61

**Anæsthetics: a Stimulus to Voluntary Effort.** By a Lady of Title - 62

**Half a Million of Money in Sight.** By Sir Rutherford Alcock, K.C.B. - 62

**The Ventilation of Hospitals.** By C. Theodore Williams, M.A., M.D., F.R.C.P. - 63

**Questions and Answers.** Wanted, a House suitable for a Convalescent Home.—One-year Probationers.—The Museum at the Royal College of Surgeons.—Varnished Paper for Hospital Walls - 65

**The National Pension Fund** - 66

**The Time of Lenten Lilies.** By a Cumberland Lady - 66

**Woodhall Spa** - 66

**The Children's Corner.**—Puzzles, Answers, etc. - vi

**Amusements with Profit** - vi

